# Synthesis and Spectroelectrochemical Investigation of Anodic Black TiO_x_ Nanotubes

**DOI:** 10.3390/nano13050931

**Published:** 2023-03-03

**Authors:** Sebastian Kotarba, Grzegorz D. Sulka, Karolina Syrek

**Affiliations:** Department of Physical Chemistry & Electrochemistry, Faculty of Chemistry, Jagiellonian University, Gronostajowa 2, 30387 Krakow, Poland

**Keywords:** black TiO_2_ nanotubes, electrochemical reduction, spectroelectrochemical measurements, optical band gap

## Abstract

Anodic TiO_2_ nanotubes were transformed into anatase at 400 °C for 2 h in air and subjected to electrochemical reduction at different conditions. It revealed that the reduced black TiO_x_ nanotubes were not stable in contact with air; however, their lifetime was considerably extended to even a few hours when isolated from the influence of atmospheric oxygen. The order of polarization-induced reduction and spontaneous reverse oxidation reactions were determined. Upon irradiation with simulated sunlight, the reduced black TiO_x_ nanotubes generated lower photocurrents than non-reduced TiO_2_, but a lower rate of electron-hole recombination and better charge separation were observed. In addition, the conduction band edge and energy level (Fermi level), responsible for trapping electrons from the valence band during the reduction of TiO_2_ nanotubes, were determined. The methods presented in this paper can be used for determination of the spectroelectrochemical and photoelectrochemical properties of electrochromic materials.

## 1. Introduction

Titanium(IV) oxide (TiO_2_), is a semiconductor that has been explored for decades as an efficient material for high-capacity batteries [1], capacitors [2], in photocatalytic decomposition of pollutants [3], solar cells [4], and in photocatalytic water splitting [5,6,7]. This broad spectrum of applications results from unique material properties such as high chemical stability, non-toxicity, low cost, commercial availability, and high resistance to photocorrosion. TiO_2_ can be synthesized in a variety of different nanostructures such as nanoparticles, nanotubes, nanopores, and nanowires [7]. Such materials offer a high surface area in relation to their mass and allow many possibilities for their modification [7]. There are many methods for the synthesis of such systems: sol-gel, hydrothermal, solvothermal, chemical or physical vapor deposition, and electrochemical methods, including anodization [7,8,9]. Anodization, i.e., the electrochemical oxidation of the metal surface, is widely used due to its high yield, simplicity, and low cost. It typically involves the formation of vertically arranged titanium(IV) oxide nanotubes on the surface of metallic titanium, and it is carried out in aqueous or organic solutions (e.g., ethane-1,2-diol, propane-1,2,3-triol) containing fluoride anions. By adjusting experimental conditions such as electrolyte composition and pH, applied voltage, and temperature, it is possible to control the diameter of the resulting nanotubes, in the range of 20–300 nm, as well as the thickness of the resulting oxide layer, up to 1 mm [9].

A characteristic feature of TiO_2_ is its ability to absorb UV radiation. It results from the band gap of this oxide, which is equal to 3.2 and 3.0 eV for anatase and rutile, respectively [7,10,11]. When a photon of energy equal to or higher than the energy of the semiconductor band gap is absorbed, an electron is transferred from the valence band to the conduction band. Photoinduced holes can easily react with the hydroxyl group chemisorbed on the oxide surface and form the hydroxyl radical ·OH, while electrons in the conduction band react with adsorbed oxygen to form the superoxide anion radical ·O_2_^−^. Both radicals are responsible for the oxidation of organic substances or the decomposition of water [2]. However, the recombination of photoinduced electrons and holes can occur very rapidly leading to the dissipation of absorbed energy in the form of heat [12]. A relatively high band gap energy of TiO_2_ limits the possibility of using naturally occurring sunlight, as the percentage of UV light reaching the Earth’s surface is about 7% [6]. To enable the effective use of titanium(IV) oxide in wide-range light-harvesting photocatalytic processes, methods such as doping with metals [7,12,13,14] or non-metals [7], deposition of metals particles on the material surface (which improves charge separation through a Schottky barrier) [3], sensitization with dyes [7,15], and the formation of heterojunctions [16] with semiconductors having lower excited interval energies are used.

Over recent years much attention was devoted to reduced black TiO_2_, which, as presented by Chen et al. [17], has a lower band gap and higher electrical conductivity. The consequence of this is the absorption of lower-energy photons, from visible light to near-infrared, resulting in an oxide color appearance that is usually black, but blue and green colors have also been reported [4,5,11,17,18,19,20,21,22]. The synthesis of black TiO_2_ typically involves a partial replacement of titanium(IV) sites with titanium(III) sites [2,15,17,19]. The semiconductor doped in this way is an n-type semiconductor, which forms a donor level just below the conduction band. Electrons from the donor level can move into the conduction band at a low energy cost and participate in reactions [23,24,25]. The synthesis of black TiO_2_ can be carried out using methods such as reduction in a high-pressure H_2_ atmosphere using a catalyst [3,5,21], reduction with aluminum (thermite mixture) [26], chemical reduction with either CaH_2_ or NaBH_4_ [2,25], high-energy proton implantation [6], and electrochemical reduction [4,11,17,18,19]. The last approach is particularly interesting as it does not require specialized equipment, and the process is easy to perform. Firstly, the polished titanium foil is anodized to obtain amorphous TiO_2_ nanotubes; then, the material is transformed into the crystalline form (e.g., anatase) by thermal annealing, after which the obtained oxide is electrochemically reduced to black TiO_x_ nanotubes. It is worth mentioning that the materials obtained in this way are often characterized by low stability [4], and therefore their improved properties compared to ordinary TiO_2_; i.e., among others a lower band gap and higher conductivity quickly (within minutes or hours) pass away after the removal of the cathodic polarization [27]. However, Song et al. [28] reported that dynamics of the ionic intercalation into/from TiO_2_ strongly depend on the viscosity of the electrolyte, so by selecting an electrolyte with an appropriate viscosity, the lifetime of black TiO_2_ can be significantly extended [4,17,18].

Spectroelectrochemical methods allow simultaneous measurement of reflectance during electrochemical polarization. Various electrochemical methods, such as linear sweep voltammetry or chronoamperometry, can be combined with spectroscopic methods to obtain for instance the UV–Vis reflectance spectra. This allows the observation of changes in the absorption properties of a sample and allows the gaining of qualitative and quantitative information about the material when it is subjected to electrical polarization. All the theoretical basis for the Kubelka-Munk transformation is discussed in the Appendix A [23,29,30,31,32,33,34,35]. For example, it is possible to collect information on the kinetics of the reaction and additional energy levels in the band gap created by semiconductor doping [36,37,38,39].

In this work, we focused on obtaining a stable layer of black anodic titanium(IV) oxide nanotubes by electrochemical reduction and investigating their spectroelectrochemical and photoelectrochemical properties. A particularly important aspect of this research was to study the influence of the reduction process on the optical band gap of black titanium oxide nanotubes and to determine the kinetics of electroreduction and succeeding oxidation reactions.

## 2. Materials and Methods

### 2.1. Electrochemical Synthesis of TiO_2_ Nanotubes

Titanium foil (99.5% purity and 0.25 mm thickness, Alfa Aesar) specimens (1 cm × 2 cm and 0.8 cm × 6 cm) were degreased in acetone and ethanol and then dried in air. Afterward, Ti coupons were electropolished in a mixture (volume ratio of 60:15:25) of acetic acid (98%, Warchem), sulfuric acid(VI) (98%, Warchem), and hydrofluoric acid (40%, Warchem) for 1 min at a constant current density of 140 mA/cm^2^ and a temperature of 20 °C. In the next step, chemical polishing was carried out in a mixture with a 1:3 volume ratio of hydrofluoric acid (40%, Warchem) and nitric acid(V) (65%, Warchem) for 10 s. Then, Ti samples were rinsed with distilled water and ethanol and then dried in air [40]. Before anodization, specimens were coated with an acid-resistant paint to select the working surface area of 1 cm × 1 cm and 0.8 cm × 1.3 cm for the 1 cm × 2 cm and 0.8 cm × 6 cm samples, respectively. Anodic oxidation of Ti was carried out using a 3-step procedure in stirred (200 rpm) ethylene glycol (Chempur, Poland) containing 0.38 wt.% NH_4_F (Sigma-Aldrich, Burlington, MA, USA) and 1.79 wt.% H_2_O at 20 °C. The anodization process was performed at 40 V for 3 h (for 1st and 2nd steps) in a two-electrode system, in which a polished Ti specimen and an as-received Ti sheet were used as an anode and cathode, respectively. After each stage, the oxide layer was removed by exfoliation using adhesive tape. The third anodizing step was carried out by applying a voltage of 40 V for 10 min in a freshly prepared solution [9]. After anodization and rinsing with water, the acid-resistant paint layer was removed.

To transform amorphous anodic titanium(IV) oxide into a crystalline anatase phase, annealing in a muffle furnace (FCF 5SHM Z, Czylok, Poland) at 400 °C (heating speed of 2 °C/min) was performed for 2 h in air. Next, a process of their activation was performed by short anodization in the anodizing electrolyte at 4 V for 10 min [4].

### 2.2. Electrochemical Reduction of TiO_2_ Nanotubes

Anodized and annealed samples were coated with the acid-resistant paint once again, to select the same working area as before annealing, and electrochemical reduction of anodic TiO_2_ was performed in a two-electrode cell at different conditions (Table 1, sample A–E). The anodized samples served as the cathode, and the platinum mesh was used as an anode. After the electrochemical reduction, the sample labeled E was protected against oxygen from the air by immersing them immediately in pure glycerol.

**Figure 1:**
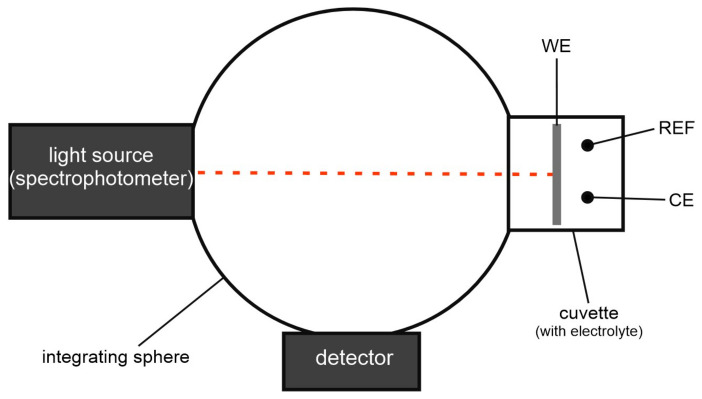
Spectroelectrochemical measurement set up with a three-electrode cell (WE—working electrode, REF—reference electrode, CE—counter electrode).

Alternatively, the activated TiO_2_ samples were electrochemically reduced in situ in the apparatus used for spectroelectrochemical measurements performed in a polystyrene cuvette using a two-electrode (TiO_2_ sample subjected to reduction served as a cathode and Pt wire as an anode) or three-electrode system (with the additional reference electrode Ag/AgCl (3.5 M KCl)) as presented in Figure 1, at the conditions denoted as F (Table 1).

The morphology of the annealed and reduced TiO_2_ nanotubes was studied by using a field emission scanning electron microscope (FE-SEM/EDS, Hitachi S-4700 with a Noran System 7, Tokyo, Japan) with an acceleration voltage of 20 kV. The crystal structure was characterized by an X-ray diffractometer (Rigaku Mini Flex II, Tokyo, Japan) with monochromatic Cu Kα radiation (λ = 1.5418 Å) at 20–60° 2θ range with a step size of 0.005° at a rate of 0.1°/min.

### 2.3. Spectroelectrochemical and Photoelectrochemical Measurements

A PalmSens4 (PalmSens BV, The Netherlands) potentiostat coupled with a Perkin Elmer Lambda 750S UV-Vis/NIR spectrophotometer (Waltham, MA, USA) equipped with an integrating sphere module was used for spectroelectrochemical measurements (Figure 1). UV–Vis diffuse reflectance spectra of the samples were recorded in the range of 300–800 nm with a step size of 1 nm at room temperature. Since the spectroelectrochemical measurements were carried out simultaneously during the in situ reduction of TiO_2_ and the solution used for it contained fluoride ions, a two-electrode system was used, where the TiO_2_ sample and Pt wire served as a cathode and anode, respectively. The chronoamperometric curves for the applied cell voltage of 8 V were recorded during in situ TiO_2_ reductions and for open circuit conditions (open circuit voltage, OCV).

Spectroelectrochemical measurements at the constant wavelength of 780 nm were also performed in the same three-electrode system, which was used for the photoelectrochemical tests (see below). Those spectroelectrochemical tests were conducted in the solution of 0.1 mol/dm^3^ LiClO_4_ (Ferak Berlin) in acetonitrile. During these measurements, the reflectance over time and linear sweep voltammograms with the scan rate of 0.5 mV/s in the range from 0 V to −2 V vs. Ag/AgCl were simultaneously recorded for the black TiO_x_ nanotubes (TiO_2_ nanotubes previously in situ electroreduced at 8 V for 15 min).

The photoelectrochemical tests on reduced TiO_2_ samples were performed directly after the in situ reduction process. The samples were transferred as quickly as possible to a quartz cuvette used as a three-electrode cell. The in situ reduced TiO_2_ nanotubes were used as a working electrode, a silver chloride electrode (3.5 M KCl) as a reference electrode, and a Pt wire as a counter electrode. For photoelectrochemical experiments, a 0.1 mol/dm^3^ solution of glycerol with KNO_3_ in a volume ratio of 3:1 was used as an electrolyte. The working electrode was illuminated with a solar simulator (power density of 100 mW/cm^2^) equipped with a high-pressure xenon arc lamp and AM 1.5 G filter (Instytut Fotonowy, Poland) combined with the PalmSens4 potentiostat. During solar light illumination, chronoamperometric curves were recorded at an open circuit potential (OCP). For the non-reduced TiO_2_ samples, OCP was measured for 30 min before starting the illumination process. For the reduced TiO_2_ samples, each subsequent chronoamperometric test started with the OCP potential determined just before starting the test.

## 3. Results and Discussion

**Figure 2:**
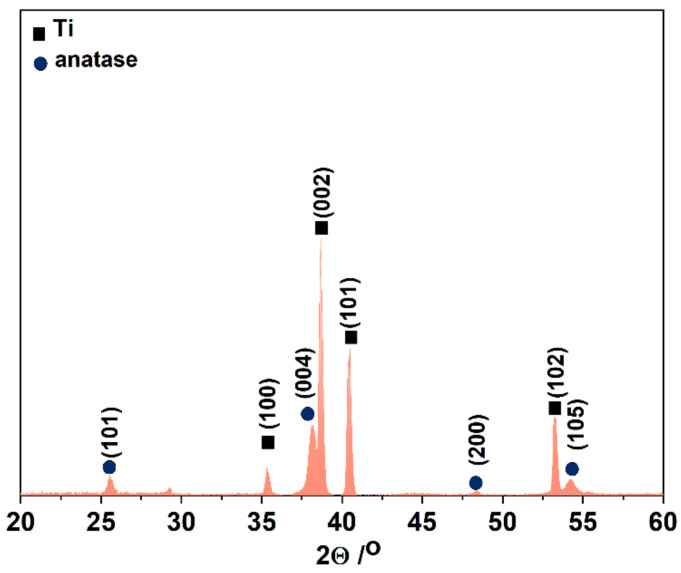
XRD pattern of anodic TiO_2_ annealed at 400 °C for 2 h in air.

The phase composition of annealed anodic TiO_2_ sample before electroreduction was investigated by X-ray diffraction (XRD). The XRD patterns (Figure 2) exhibited two components. The main diffraction peaks correspond to the reflection from (1 0 0), (0 0 2), (1 0 1), (1 0 2) titanium (JCPDS card no. 05-0682) and (1 0 1), (0 0 4), (2 0 0), (1 0 5) anatase (JCPDS card no. 21-1272) crystal planes.

**Figure 3:**
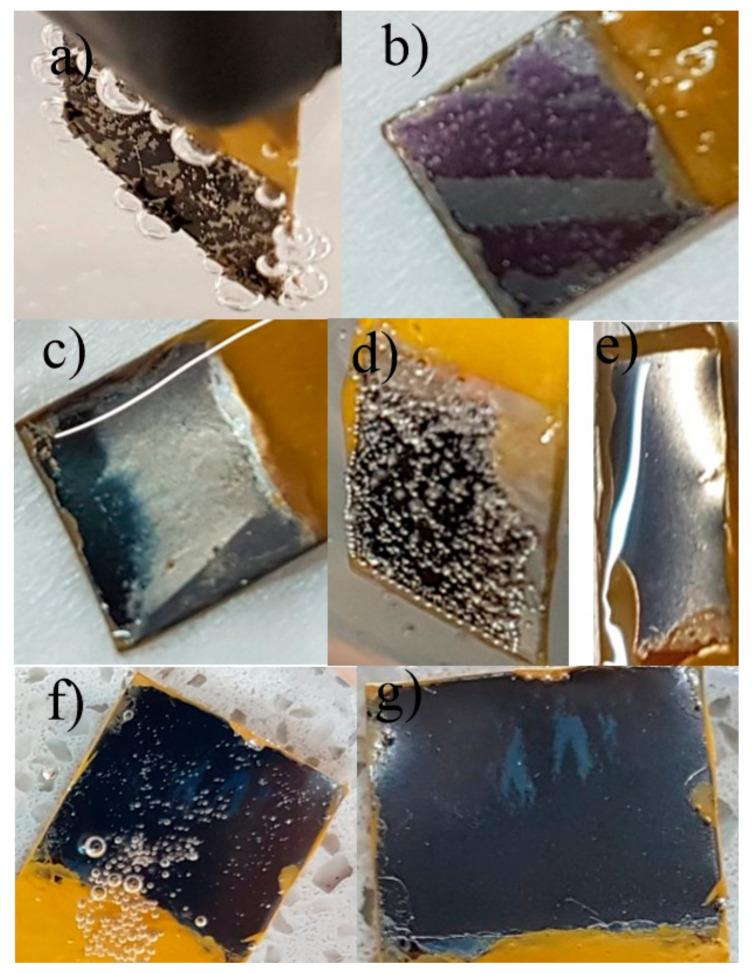
Macroscopic images of the TiO_2_ samples electrochemically reduced at different conditions: (**a**) sample A during the reduction at 5 V for 60 min in a 1:1 glycerol–water solution with 0.27 wt.% NH_4_F, (**b**) sample B reduced at 5 V for 5 min in a 1:1 glycerol–water solution with 0.27 wt.% NH_4_F after its removal from the electrolyte, (**c**) sample C reduced at 5 V for 5 min in a 3:1 glycerol–water solution with 0.13 wt.% NH_4_F after its removal from the electrolyte, (**d**) sample E during the reduction at 5 V for 10 min in a 50:1 glycerol–water solution with 0.27 wt.% NH_4_F, (**e**) reduced sample E after 5 min from its removal from the electrolyte, (**f**) reduced sample E immediately after its immersion in glycerol, and (**g**) reduced sample E after a 1.5 h immersion in glycerol.

The appearances of typical anodic TiO_2_ samples reduced at the conditions listed in Table 1 are shown in Figure 3. Although black TiO_2_ nanotubes were observed during the reduction for sample A (Figure 3a), a long reduction time favored the evolution of gaseous hydrogen in a competitive reaction and caused damage to the nanotube oxide layer, which was manifested by its exfoliation from the surface of the titanium foil. A positive result of the electrochemical reduction, demonstrated by the darkening of the surface upon the applied voltage, disappeared within about 5 min after the sample removal from the electrolyte. In the next approach (sample B, Figure 3b) similar reduction conditions were applied except the reduction time, which was reduced to 5 min to avoid the oxide layer exfoliation by evolving hydrogen gas. Nevertheless, the black color disappeared also after 5 min.

Samples C and D were reduced in the electrolyte having higher viscosity (a 3:1 glycerol-H_2_O solution with 0.13 wt.% NH_4_F) for 5 min at 5 V and 2 V, respectively. For sample C (Figure 3c), darkening of the oxide surface and a lack of exfoliation of nanotubes from the Ti surface were observed. However, as the sample was removed from the electrolyte, the black layer flowed down towards the edge of the sample, and similarly, as for the previous samples, the color quickly disappeared. For the TiO_2_ sample (sample D) reduced at 2 V for 5 min, no color change in the sample was observed during the reduction process.

Next, electroreduction tests of TiO_2_ nanotubes were carried out at 5 V for 10 min in an electrolyte with even higher viscosity, i.e., a 50:1 (in vol.) glycerol-water solution with 0.27 wt.% NH_4_F (sample E). Initially, promising results were observed, as the sample darkened noticeably during the electrochemical reduction, and the black layer did not flow down after the removal of the sample from the electrolyte (Figure 3d); however, the black color disappeared quickly when the sample was exposed to air (Figure 3e). Since the reduced black TiO_2_ nanotubes oxidized very quickly when they came in contact with air and since this process affects the reversibility of TiO_2_ reduction to TiO_x_, we decided to limit the access of atmospheric oxygen to the oxide layer by immersion of sample E in pure glycerol (Figure 3f). A significantly prolonged black color lifetime of the reduced oxide layer for sample E was observed even after 1.5 h immersion in glycerol (Figure 3g). These tests confirmed the effectiveness of protecting nanotubes against oxidation by immersing them in glycerol.

**Figure 4:**
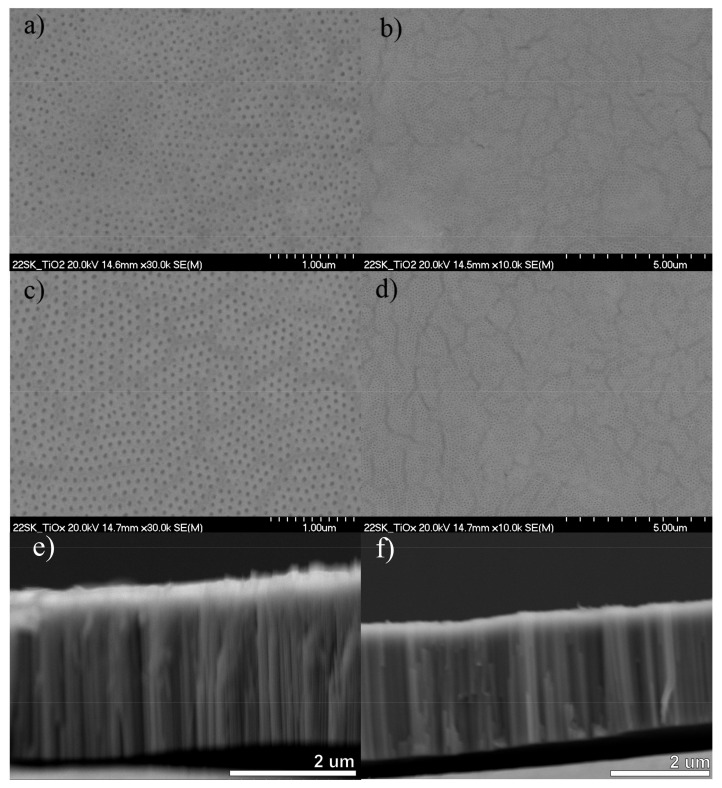
SEM images of TiO_2_ nanotubes after (**a**,**b**,**e**) anodization and annealing, and (**c**,**d**,**f**) electrochemical reduction at 5 V for 10 min in a 50:1 glycerol–water solution with 0.27% NH_4_F (sample E).

Figure 4 shows SEM top-view images of the anodic TiO_2_ nanotubes after the annealing process (Figure 4a,b) and subjected to the electrochemical reduction (similarly as the sample E) at 5 V for 10 min in a 50:1 glycerol–water solution with 0.27 wt.% NH_4_F (Figure 4c,d). As can be seen, before the electrochemical reduction anodic TiO_2_ nanotubes have a pore diameter of 49.9 ± 1.7 nm. It is demonstrated that the electroreduction process did not change the morphology of TiO_2_ nanotubes. The arrangement of nanotubes and oxide layer was not destroyed by the applied voltage.

**Figure 5:**
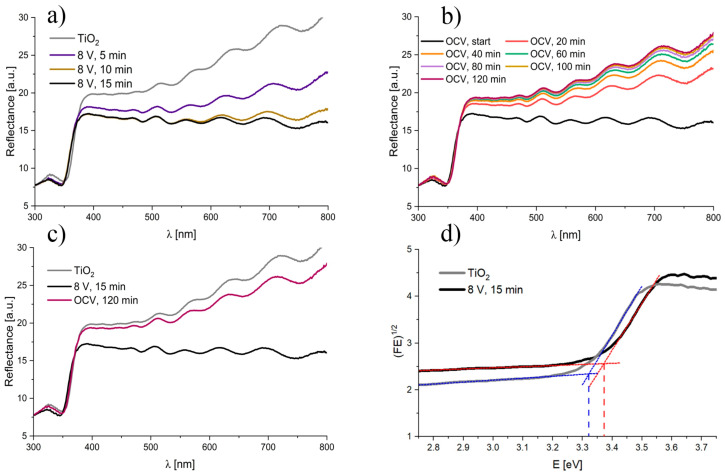
UV–Vis diffuse reflectance spectra recorded (**a**) before and during the cathodic reduction of TiO_2_ nanotubes at 8 V, (**b**) during OCV conditioning after the completion of the electroreduction of TiO_2_ nanotubes, (**c**) for the sample before and after electroreduction for 15 min at 8 V, and after 120 min at OCV conditions. Tauc plots for (**d**) TiO_2_ before and after reduction at 8 V for 15 min.

To exclude the problem of transferring an unstable sample from the electrochemical cell to pure glycerol and then to a PS cuvette filled with a suitable solution for spectroelectrochemical measurements, we decided to perform the electroreduction of TiO_2_ nanotubes directly in the spectroelectrochemical setup. As no changes were observed in the UV–Vis diffuse reflectance spectra when small reduction voltages were applied (e.g., 0.2–5 V), the cathodic potential of 8 V for 15 min was applied to efficiently reduce TiO_2_ nanotubes in a glycerol-H_2_O solution (vol. 50:1) with 0.27 wt.% (sample F in Table 1). During the reduction process UV–Vis, diffuse reflectance spectra were recorded (Figure 5a), and after its completion, the open-circuit voltage (OCV) was measured for 120 min (Figure 5b). To ensure that the used measuring system did not affect the results, the reflectance spectrum of the solution itself was measured in the same cuvette used for the measurements, against the BaSO_4_ layer as a background. As can be seen in Figure 5a, reflectance of the tested sample changed significantly during the reduction process. The reduced TiO_2_ showed significantly lower reflectance in the entire spectral range (380–800 nm); however, larger differences between non-reduced and reduced TiO_2_ could be observed at higher wavelengths. After reducing the nanotubes, when no cathodic potential was applied to the sample and when the circuit was open (OCV), reverse changes in reflectance over time were observed (Figure 5b). The longer the time after the reduction was completed, the higher the reflectance was, which suggests the reversibility of the reduction of TiO_2_ nanotubes (see a comparison in Figure 5c). The optical band gap determined from the Tauc plots is equal to 3.32 eV and 3.37 eV for TiO_2_ before and after the reduction process, respectively (Figure 5d). Those values are in agreement with the literature reports. For instance, it was reported by Nair et al. [41] that Ti^3+^ defects can be characterized by the band gap energy of 2.52 eV, Ti^3+^ and oxygen vacancies 2.8 eV, and black TiO_2_ 1.85 eV or 2.44 eV [42,43,44].

The observed waves in the 500–800 nm region might originate from the Fabry–Pérot oscillations. However, Song et al. [45] reported that the appearance of such an effect is connected to the morphology of the tube walls. It was shown that in the case of single or double-layer smooth tube walls, only a broad reflection peak is present. To achieve Fabry–Pérot fingers, rippled tubes have to be formed by adding more water into the ethylene glycol-based electrolyte. Anodic TiO_2_ synthesized in this paper had smooth walls and a total thickness of ~2.5 ± 0.15 µm (see Figure 4). Nevertheless, as this effect is observed for the nonreduced material and above 800 nm it is a physical phenomenon.

**Figure 6:**
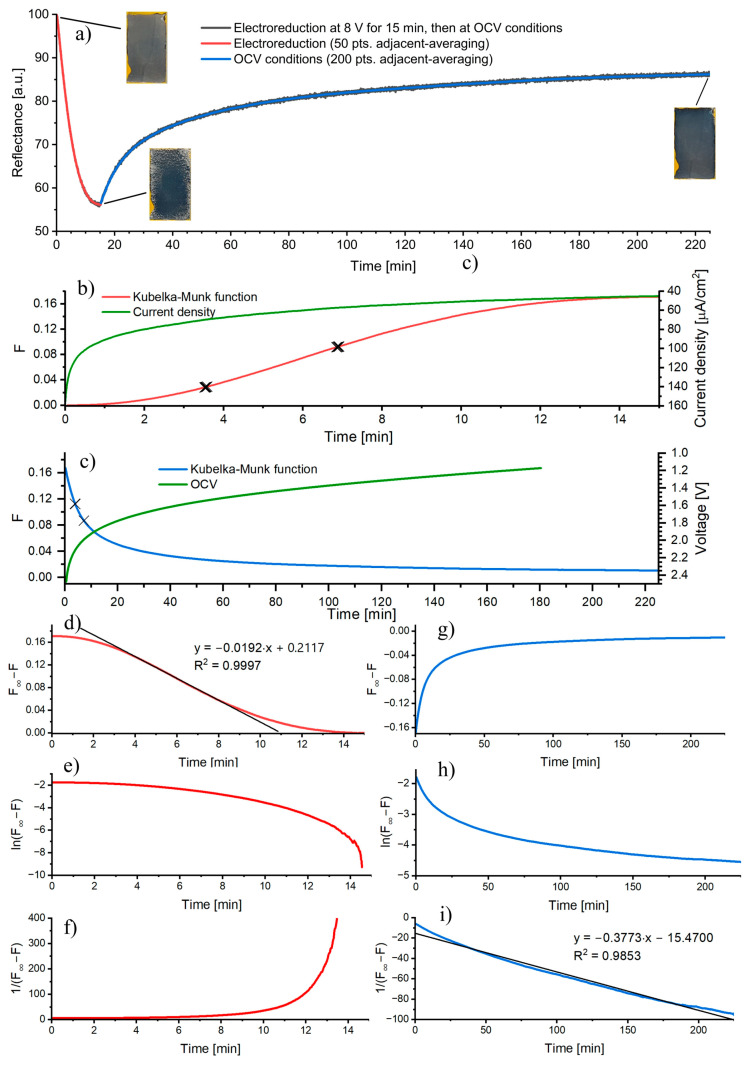
Reflectance vs. time curve (**a**) for the TiO_2_ sample subjected to the electrochemical reduction at 8 V for 15 min and then conditioned in the solution at OCV conditions for 4 h. Insets: macroscopic images of the studied sample surface at various stages of the process. The Kubelka–Munk function and current density as functions of time (**b**) for the electroreduction process. The Kubelka–Munk function and potential as functions of time (**c**) for the sample conditioned at OCV conditions. Graphs of a zeroth-order (**d**,**g**), first-order (**e**,**h**), and second-order (**f**,**i**) reactions for the TiO_2_ electroreduction (**d**–**f**) and oxidation of reduced TiO_2_ by air oxygen (**g**–**i**).

Since the largest changes in the reflectance spectrum were observed at the wavelength of 750–800 nm for the reduction of TiO_2_ at 8 V for 15 min (see Figure 5c), the reflectance at the wavelength of 780 nm was measured continuously during the electroreduction process and then during conditioning at OCV for 4 h (Figure 6a). For this measurement, the reference reflectance was set to be the starting reflectance of the sample; hence, R = 100% at t = 0. The experimental values were averaged using the 50 pts. and 200 pts. adjacent-averaging function (OrginLab) for the electroreduction and OCV conditioning, respectively. The resulting data were converted to the Kubelka–Munk function and presented in Figure 6b,c. For the TiO_2_ electroreduction at 8 V (Figure 6b), the Kubelka–Munk function increases, while at the OCV conditions decreases. As expected, the current density recorded during the reduction decreases with time and reaches a plateau after 10 min which is related to undergoing reduction of the nanotubular layer. On the other hand, at open circuit conditions, the potential recorded for 3 h decreases from ca. 2.5 V to 1.15 V (Figure 6c), indicating a gradual oxidation with air oxygen (most probably Ti^3+^ ions are oxidized to Ti^4+^ ions). However, the reduced TiO_2_ sample retains its black color after 4 h of conditioning at open circuit conditions (insets in Figure 6a).

Knowing that the Kubelka–Munk function depends linearly on the concentration (Equation (A4), Appendix A) and scattering coefficients of both TiO_2_ forms (non-reduced and reduced), are very similar as no visible changes in the oxide morphology are observed; the kinetics of TiO_2_ electroreduction and oxidation by air oxygen can be derived from the reflectance measurements. To determine the order of the reaction, the characteristic graphs for the zeroth-, first-, and second-order reactions were plotted for both processes, the TiO_2_ electroreduction (Figure 6d–f) and oxidation of the reduced TiO_2_ by air oxygen (Figure 6g–i). Under studied conditions, the reduction reaction obeys the zero-order kinetics (Figure 6d), and the reaction rate does not depend on the concentration of Ti^4+^ ions in the oxide lattice, while the oxidation reaction obeys the second-order kinetics (Figure 6i). This suggests that the reaction rate is rather proportional to the concentrations of reactants (Ti^3+^ and oxygen). The reaction order calculated using the differential van’t Hoff method (Equation (A6), Appendix A), was $n_{red.}=0.05$ and $n_{ox.}=2.21$ for the reduction and oxidation, respectively. The points used for the calculations were marked with crosses in Figure 6b,c. It is worth noticing that both the graphical and van’t Hoff methods gave similar results.

**Figure 7:**
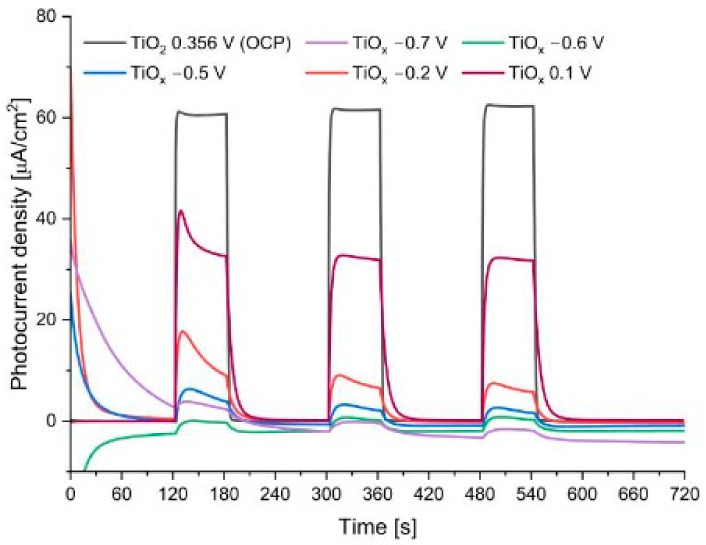
Photocurrent density vs. time curves under chopped simulated sunlight illumination for non-reduced TiO_2_ and reduced TiO_x_ samples at various OCP potentials.

To limit the reversibility of the reduction–oxidation reaction during photoelectrochemical measurements with the reduced black TiO_x_ nanotubes, a glycerol solution with 0.1 mol/dm^3^ KNO_3_ in a volume ratio of 3:1 was used as an electrolyte. The three-electrode system with the Ag/AgCl reference electrode was used. To avoid any differences in TiO_2_ morphology, the photoelectrochemical tests were performed for the same TiO_2_ sample before and after the electroreduction process performed in situ at 8 V for 15 min. As the OCP value did not stabilize during the open-circuit potential measurement, most likely due to the continuous oxidation of Ti^3+^ to Ti^4+^ in the lattice of black TiO_x_ nanotubes, the sample was polarized by applying the OCP potential determined immediately before each measurement. The resultant photocurrent density generated during the exposure to simulated sunlight as a function of time is shown in Figure 7 for different potentials (OCPs) applied in the consecutive measurements in the following order −0.7 V, −0.6 V, −0.5 V, −0.2 V, and 0.1 V vs. Ag/AgCl.

At polarization potentials more negative than −0.5 V vs. Ag/AgCl, the dark current density (light off) took negative values with time. For the polarization potential of −0.2 V vs. Ag/AgCl and more positive, no significant dark current densities were observed; however, the generated photocurrent (light on) was very low. Therefore, after the measurement at −0.2 V vs. Ag/AgCl, the sample was conditioned without polarization in the solution for 3 h. Then, another photoelectrochemical test was performed at the measured OCP potential, i.e., 0.1 V vs. Ag/AgCl. The photoresponse was significantly higher than those for the previous measurements carried out for this sample but still was lower than that for the non-reduced TiO_2_ nanotubes. This difference may result from the applying different polarization potentials; however, for unstable black TiO_2_, when a higher potential was applied, the material oxidized itself.

**Figure 8:**
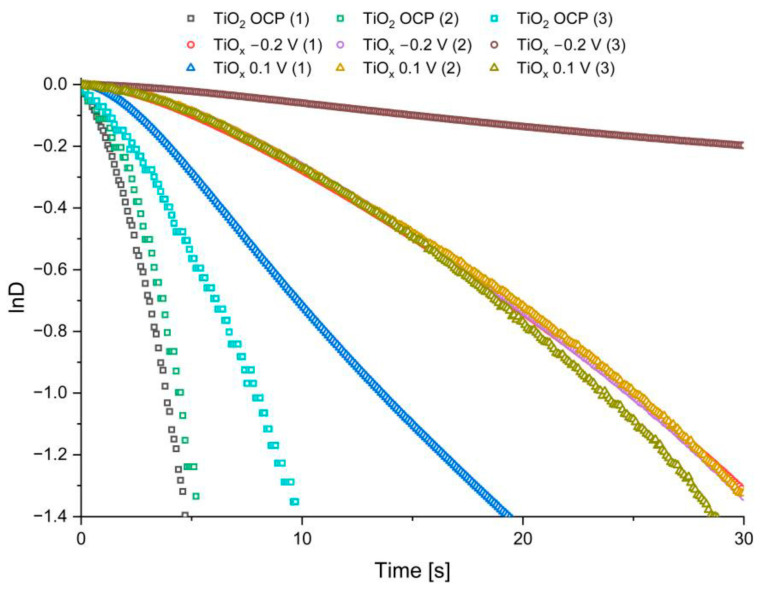
ln*D* vs. time dependence for the non-reduced and reduced TiO_2_ polarized by applying different OCPs. Number in brackets indicates the number of lamp shutter opening.

In addition, based on Radecka et al. [14], the kinetics of the hole-electron recombination occurring in the studied TiO_2_ material was determined using the following equation:

(1) $$D=exp\left(-\frac{t}{\tau }\right),$$

at which *D* is defined as:

(2) $$D=\frac{I\left(t\right)-I\left(f\right)}{I\left(i\right)-I\left(f\right)},$$

where 𝑡 is time; 𝜏 is the time constant of the transition state; 𝐼 is the photoanode or photocathode current; 𝑖 refers to the initial state; and 𝑓 refers to the final steady state. By plotting ln𝐷 against time, we can determine the time constant by fitting a straight line, which slope represents the pseudo-first-order constant. Since ln𝐷 vs. time data for different consecutive shatter openings (Figure 8) deviate strongly from a linear relationship, a complex reaction mechanism is highly probable. Therefore, based on Hagfeldt et al. [46] and Tafalla et al. [47] we read 𝜏 values from the graphs for ln*D* = −1. The obtained results are collected in Table 2. As can be seen, the time constant values are significantly higher for the reduced TiO_2_, which indicates a slower rate of recombination of hole-electron pairs and better charge separation.

**Figure 9:**
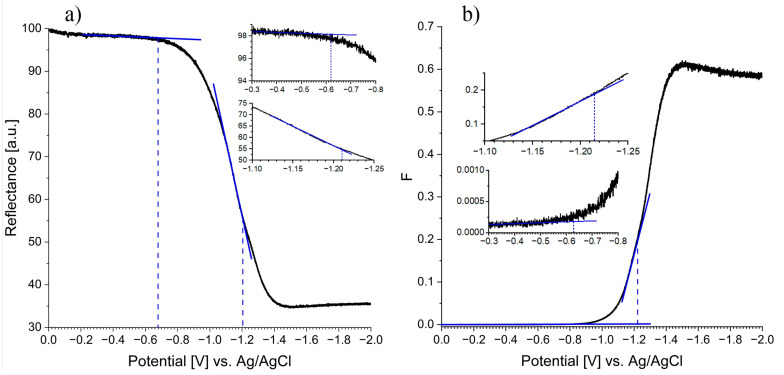
Reflectance at 780 nm (**a**) and Kubelka–Munk function (**b**) vs. polarization potential for the reduced black TiO_x_ nanotubes. The electrode was polarized from 0 V to −2 V vs. Ag/AgCl with a scan rate of 0.5 mV/s.

It is widely known that Ti^3+^ centers in reduced TiO_x_ are responsible for the formation of the broad peak in the absorption spectrum, with maximum localized at ca. 780 nm [48]. To characterize the redox properties of the synthesized black TiO_x_ nanotubes, spectroelectrochemical measurements were performed at 780 nm. Since the reflectance of the black TiO_x_ nanotubes over time and linear sweep voltammetry (LSV) were measured simultaneously, it was possible to plot the dependence of the reflectance or Kubelka–Munk function (they can be used interchangeably) on the polarization potential (Figure 9). It is widely recognized that by a polarization of the electrode towards more negative values, the Fermi level of the electrode changes, and the conduction band starts to be reduced. Consequently, the inflection point (at −0.62 V vs. Ag/AgCl) located at the potential at which reflection decreases (Fermi level) corresponds to the process when electrons from the valence band are trapped at energy levels in the band gap. On the other hand, the inflection point at a more negative potential (at −1.21 V vs. Ag/AgCl), before the function ‘flattens out’, corresponds to the edge of the conduction band.

The determined energy level in the band gap as well as the edge of the conduction band was compared with the results presented in Buchalska et al. [39] for nanocrystalline anatase. While the edge of the conductivity band determined here (−1.21 V vs. Ag/AgCl) is in good agreement with the previously published potential of −1.14 V vs. Ag/AgCl, the determined Fermi level is shifted towards a more negative potential (−0.62 V vs. Ag/AgCl) compared to the previously reported −0.41 V vs. Ag/AgCl. This is most likely due to the presence of oxygen in the solution, which can react with the resulting Ti^3+^ centers according to the equation [38]:

(3) $$Ti^{III}+O_{2}\to Ti^{IV}+O_{2}^{\cdot -}$$

Although the solution was bubbled with argon for 15 min before the measurement, during the spectroelectrochemical test the Ar atmosphere was not maintained over the solution, which may affect the measuring system [38,39].

## 4. Conclusions

In the present work, anatase TiO_2_ nanotubes were successfully obtained by anodization followed by annealing. The black TiO_x_ nanotubes were obtained by electrochemical reduction at different conditions; however, they rapidly oxidized in contact with oxygen air. The oxidation process was significantly retarded by immersing the TiO_x_ nanotubes in glycerol, which plays the role of a medium protecting the material from atmospheric oxygen. Consequently, the black color of reduced samples remains even for a few hours. Spectroelectrochemical measurements allow for determining the optical band gap of the non-reduced and electroreduced oxides (3.32 and 3.37 eV, respectively). It was found using the graphical and differential van’t Hoff methods that the polarization-induced reduction of TiO_2_ and spontaneous oxidation of reduced TiO_x_ by oxygen from air obey zeroth- and second-order kinetics, respectively. The photoelectrochemical measurements revealed that the reduced TiO_x_ generated lower photocurrents during exposure to simulated sunlight compared to non-reduced TiO_2_, but its higher recombination time constant indicates a lower rate of electron-hole recombination under experimental conditions. The determined electron-trapping energy level (Fermi level) for the reduced anodic TiO_x_ was −0.62 V vs. Ag/AgCl.

## Figures and Tables

**Table 1:** Experimental conditions applied for electrochemical reduction of anodized TiO_2_ nanotubes.

Sample Label	Electrolyte	Voltage (V)	Time (min)
A	Glycerol-H_2_O (vol. 1:1) + 0.27 wt.% NH_4_F	5	60
B	Glycerol-H_2_O (vol. 1:1) + 0.27 wt.% NH_4_F	5	5
C	Glycerol-H_2_O (vol. 3:1) + 0.13 wt.% NH_4_F	5	5
D	Glycerol-H_2_O (vol. 3:1) + 0.13 wt.% NH_4_F	2	5
E	Glycerol-H_2_O (vol. 50:1) + 0.27 wt.% NH_4_F	5	10
F	Glycerol-H_2_O (vol. 50:1) + 0.27 wt.% NH_4_F	8	15

**Table 2:** Transition state time constant for the non-reduced and reduced TiO_2_ polarized at different OCPs for ln*D* = −1.

Shutter Opening Number	*τ* (s)		
	TiO_2_	TiO_x_ (at 1.0 V)	TiO_x_ (at −0.2 V)
1	3.86	13.67	24.88
2	4.51	25.04	24.87
3	7.88	23.76	-

## Data Availability

Not applicable.

## Appendix A

To characterize the optical properties of solids by UV–Vis diffuse reflectance spectroscopy (DRS) the Kubelka–Munk model can be used. For transmission spectroscopy, where a beam of light is passed through a sample, the transmittance is expressed as follows [29]:

(A1) $$T=\frac{I}{I_{0}},$$

where T is the transmittance (takes the value 1 for a completely transparent sample); I is the intensity of light after passing through the sample; and $I_{0}$ is the intensity of light incident on the sample. Similarly, for diffusion reflectance spectroscopy, reflectance is expressed by the following equation [29]:

(A2) $$R_{\infty }=\frac{J}{I_{0}},$$

where $R_{\infty }$ is the absolute reflectance (for a sample that is infinitely thick, there is no reflectance from the substrate); J is the intensity of the reflected light; and $I_{0}$ is again the intensity of the incident light. A perfectly reflective substance would have $R_{\infty }=1$. Since it is impractical to measure absolute reflectance, as for transmittance, the value usually measured is a relative reflectance [29]:

(A3) $$R_{\infty }^{\prime }=\frac{R_{\infty \;sample}}{R_{\infty \;standard}},$$

where the standard could be, for example, BaSO_4_.

In transmission spectroscopy, it is often convenient to represent data in units of absorbance A, where A=log(1/T); according to the Beer’s law, the concentration of a substance is directly proportional to its absorbance. It is possible to plot a similar function for reflectance, but this does not imply the correctness of Beer’s law for DRS. To use a function with similar functionality to the Beer’s law for absorption spectroscopy, the function derived by Kubelka and Munk [29] should be applied:

(A4) $$F\left(R_{\infty }\right)=\frac{{\left(1-R_{\infty }\right)}^{2}}{2R_{\infty }}=\frac{K}{S}=\frac{2.303\varepsilon c}{S},$$

where *K* is the absorption coefficient; *S* is the sample scattering coefficient; *ε* is the molar absorption coefficient; and *c* is the analyte concentration [29,30,31]. It is worth mentioning that both *K* and *S* depend on the illumination geometry and, consequently, do not represent intrinsic absorption *α* and scattering s coefficients. To estimate the optical band gap of crystalline semiconductors, Tauc plots can be used, i.e., dependence between hν (photon energy) and $\alpha h\nu^{1/f}$, where f reflects the nature of the semiconductor’s band gap. For TiO_2_ with an indirect band gap, f=2 [32]. It is generally accepted that the absorption coefficient of the material ($\alpha_{KM}$) is directly proportional to the Kubelka–Munk function ($F\;\left(R_{\infty }\right)\sim \alpha_{KM}$), and this approximation is sufficient to calculate the optical band gap of a semiconductor [23,32,33,34]. Since the concentration of the substance is directly proportional to the Kubelka–Munk function (Equation (A1)), it is possible to apply the previously known methods of analyzing the concentration of the sample for diffuse reflectance spectroscopy. This is particularly useful when reaction kinetics is studied. Therefore, to determine the kinetic parameters of the process (e.g., reaction order), we can use a graphical method based on plotting appropriate graphs of the Kubelka–Munk function for the wavelength used during measurements as a function of time. Consequently, the time dependence of $F\left(R\right)-F\left(R_{i}\right)$ for zero-order reactions, $\ln\left(F\left(R\right)-F\left(R_{i}\right)\right)\;$ for first-order reactions, and $1/\left(F\left(R\right)-F\left(R_{i}\right)\right)$ for second-order reactions should be considered, and from linear relationships the reaction order can be determined. For a fractional order reaction, the van’t Hoff differential method can be used and the reaction order, *n*, equal to:

(A5) $$n=\frac{\log\left(r_{1}/r_{2}\right)}{\log\left(c_{1}/c_{2}\right)},$$

where *r* is the instantaneous reaction rate (d*c*/d*t*); c is the concentration of the reactant; and *t* is time. Using the differential method, i.e., replacing the instantaneous reaction rates by the average over a certain time interval that is Δ*c*/Δ*t* [35], and knowing that the concentration depends linearly on the Kubelka–Munk function, the following formula can be used:

(A6) $$n=\frac{\log\left(\left(\Delta F\left(R_{1}\right)/\Delta t\right)/\left(\Delta F\left(R_{2}\right)/\Delta t\right)\right)}{\log\left(\Delta F\left(R_{1}\right)/\Delta F\left(R_{2}\right)\right)}$$
